# Historical change in fish species distribution: shifting reference conditions and global warming effects

**DOI:** 10.1007/s00027-014-0386-z

**Published:** 2015-01-03

**Authors:** Didier Pont, M. Logez, G. Carrel, C. Rogers, G. Haidvogl

**Affiliations:** 1 Irstea UR HBAN, 1 rue Pierre-Gilles de Gennes-CS 10030, 92761 Antony, France; 2 Irstea UR HYAX, Pôle Onema-Irstea Hydroécologie Plans d’eau, 3275, Route de Cézanne-CS 4006, 13182 Aix-en-Provence Cedex 5, France; 3 Irstea UR HYAX, 3275 Route de Cézanne-CS 4006, 13182 Aix-en-Provence Cedex 5, France; 4 Institute of Hydrobiology and Aquatic Ecosystem Management, University of Natural Resources and Life Sciences Vienna, Max-Emanuel-Strasse 17, 1180 Vienna, Austria

**Keywords:** Historical data, Species distribution model, Fish, Climate change, Uncertainty, Reference conditions, Shifting baseline

## Abstract

Species distributions models (SDM) that rely on estimated relationships between present environmental conditions and species presence-absence are widely used to forecast changes of species distributions caused by global warming but far less to reconstruct historical assemblages. By compiling historical fish data from the turn to the middle of the twentieth century in a similar way for several European catchments (Rhône, Danube), and using already published SDMs based on current observations, we: (1) tested the predictive accuracy of such models for past climatic conditions, (2) compared observed and expected cumulated historical species occurrences at sub-catchment level, and (3) compared the annual variability in the predictions within one sub-catchment (Salzach) under a future climate scenario to the long-term variability of occurrences reconstructed during an extended historical period (1800–2000). We finally discuss the potential of these SDMs to define a “reference condition”, the possibility of a shift in baseline condition in relation with anthropogenic pressures, and past and future climate variability. The results of this study clearly highlight the potential of SDM to reconstruct the past composition of European fish assemblages and to analyze the historical ecological status of European rivers. Assessing the uncertainty associated with species distribution projections is of primary importance before evaluating and comparing the past and future distribution of species within a given catchment.

## Introduction

For decades, freshwater biodiversity has been recognized as highly threatened due to the long history of anthropogenic modifications of continental aquatic ecosystems (Dudgeon et al. 2006). Among aquatic species, fish react to almost every kind of water quality and habitat alterations (Ormerod 2003). Fish sensitivity to human pressures is a basis for using fish-based biological monitoring tools to assess environmental change (Fausch et al. 1990). More recently, numerous papers have also pointed out the influence of climate modifications on freshwater ecosystems (Webb 1996) and in particular on fish species distributions (Xenopoulos et al. 2005; Graham and Harrod 2009). Long-term changes in climate during the last millennia are well known, particularly those since the end of the little ice age in the mid-19th century (Schurer et al. 2013). Evidence is now accumulating that one of the main species responses to global warming is a shift poleward or upward in elevation to colonize favorable thermal habitats (Parmesan and Yohe 2003; Crimmins et al. 2011; Comte and Grenouillet 2013).

In both cases, human alteration and climate change, the impact of environmental modifications on species distributions is based on the comparison between an observed or a predicted situation and a benchmark. In bioassessment methods, a reference condition is determined from sites undisturbed by anthropogenic stressors, thus representing continuity with a former condition (Bailey et al. 1998). In Europe, most catchment landscapes and rivers have undergone modifications during the last two centuries. Undisturbed habitats are becoming increasingly rare and bioassessment methods generally rely on what are now judged to be minimally-disturbed sites (Stoddard et al. 2006). Moreover, even for such sites, the distributions of species could continuously evolve in response to large-scale anthropogenic disturbance, past changing thermal and rainfall conditions, and to the expected future climate trend (Tingley and Beissinger 2009). The term “shifting baseline” was developed to refer to such long-term changes over generations which are difficult to recognize (Pauly 1995).

Historical reconstruction of species distribution could allow testing such “shifting baseline” effects. Numerous studies have used printed historical information on fish communities from the late eighteenth and nineteenth centuries to reconstruct the long-term evolution of fish faunas (Rinne et al. 2005; Maceda-Veiga et al. 2010), to estimate reference conditions (Carrel 2002; Wolter et al. 2005; Winter et al. 2009) or to define specific conservation programs (Worthington et al. 2010). Different types of historical sources have been identified depending on the possibility to gain quantitative or only qualitative data at different scales (Haidvogl et al. 2013a). From the late eighteenth century on, early scientific fish ecological surveys were conducted compiling scientific inventories and systematic or selective enquiries of fishermen and fishmongers. From the second half of the nineteenth century, fish distribution maps were also produced in several catchments across Europe, localizing species occurrences in precise rivers or river sections (Haidvogl et al. 2015; Carrel 2002). As opposed to most other written sources, historical maps are among the most informative because the aim of the historical maps was to describe the complete fish fauna and not only the species of commercial interest (Haidvogl et al. 2013a).

Several authors have attempted to model historical fish species distribution. Lassalle and Rochard (2009) compiled literature on past occurrences of anadromous species to model their distribution before the twentieth century and to simulate potential change due to climate change. In a recent work, Labay et al. (2011)—using both historical and present data—applied species distribution models (SDM) to establish baseline conditions and to assess the current status of a river fish community. Such models are also widely used to predict the change of fish distributions under climate change scenarios (Austin 2007; Buisson et al. 2008). A classical approach is to model present fish distributions based on recent sampling surveys and to compare the currently forecasted distribution with the expected distribution under different climate conditions (Buisson et al. 2008).

The main aim of the present paper was to examine the ability of SDM to reconstruct historical freshwater fish assemblages and discuss the implication of using baseline conditions in the context of predicting species distribution under changing environmental conditions. The already published SDM used here are based on current observations from European rivers qualified as current “reference sites” (Logez et al. 2012).

The analyses we presented are based on historical species occurrence from two large catchments (Upper Danube, Salzach catchment: late nineteenth—early twentieth century, Rhône catchment: first half of the twentieth century). Our objectives were: (1) to compile historical data in a comparative manner for these two catchments, (2) to use the previously published SDM (Logez et al. 2012) to predict the species occurrences in the historical period and to compare them with the historical observations, (3) to compare the SDM’s performances between catchments and to analyze the capability of predicted occurrences to reflect the historical community structure within the different studied catchments, and (4) to compare the annual variability in the predictions within one sub-catchment (Salzach) under a future climate scenario to that reconstructed during an extended historical period (1800–2000). The main aim of this last point is to evaluate the potential importance of past and future climatic change on the species distributions Finally, we discussed the potential of these SDMs to define a “reference condition” and the possibility of a shift in baseline condition within the last century in relation with climatic variability and/or anthropogenic modification of the river system.

## Methods

### SDM based on current observations

We applied SDM previously published by Logez et al. (2012) to evaluate the ability of SDM, based on present data, to correctly reconstruct historical fish species distributions. These SDM were calibrated using a dataset of 1548 European sites considered as representative of reference conditions. These “minimally disturbed sites” (Stoddard et al. 2006) were selected using an explicit set of water quality and physical-habitat criteria (Pont et al. 2006). The sites were sampled using electrofishing methods either by wading or by boat during the last 30 years (1981–2007).

Four environmental variables were considered to estimate the habitat requirements of species. The upstream drainage area (UDA in km^2^) is a descriptor of the position of the stream reach along the hydrographic network (Pont et al. 2005) and also reflects habitat diversity because stream complexity increases along the longitudinal gradient (Matthews 1998). Stream power (STP, watt m^−1^) is ‘the rate of potential energy expenditure over a reach or STP per unit of stream length’ (Gordon et al. 2004). It varies with both river slope and stream discharge and reflects the power of a stream to move bed substrate and change hydraulic conditions, two environmental variables of primary importance for fish. The mean air temperature in July (TJUL, °C) was used as a proxy for water temperature, and the thermal amplitude between July and January (TDIF, °C) allowed considering the annual variability of thermal condition and the differences between oceanic and continental climates (definition of the growth period of fish). TJUL and TDIF were also chosen for their low correlation in order to limit the multicollinearity between explanatory variables. As the objective was to describe the habitat requirements of fish species “independently” from local human alterations, these SDM attempted to avoid parameters overly influenced by anthropogenic disturbances. For example, the mean annual discharge value used to estimate STP was a mean annual run-off, depending only on the annual rainfall and the annual evaporation over UDA. All the environmental variables describing climatic conditions (temperature, rainfall) were averaged over the 10 years before the sampling date in order to consider the mean climatic conditions prevailing during the life span of most of the fish species considered.

Logistic regression, a special case of generalized linear model (GLM) with binomial error distribution, is a classical statistical method and is recognized for its capability to estimate a species’ niche (Austin 2007) when the model includes quadratic terms for environmental variables. In addition, GLM enables computing confidence intervals around expected values for a future observation (CI) (Faraway 2006).

### Historical study area

**Fig. 1:**
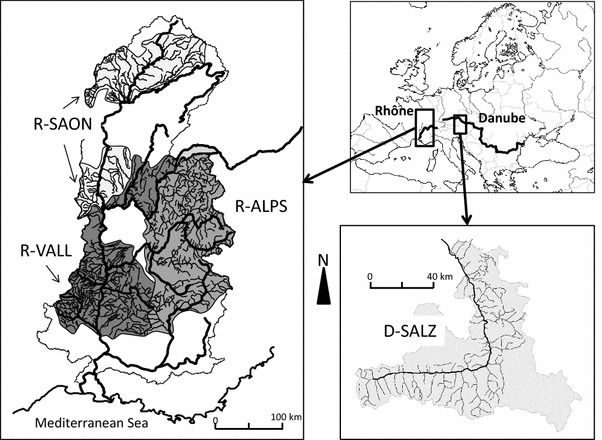
Studied areas in the Danube and Rhône catchments: Salzach River (D-SALZ), Rhône alpine tributaries (R-ALPS), Rhône valley tributaries (R-VALL) and Saône River (R-SAON)

The study area comprised river sections belonging to the Danube (D) and to the Rhône (R) catchment (Fig. 1). The Salzach River, with a total catchment area of 6,734 km^2^ (D-SALZ) is a 225-km-long tributary of the Inn River, which is itself the largest sub-catchment of the Upper Danube (251 m^3^ s^−1^ at the confluence of the Inn River). The discharge regime is snow-dominated but with glacial influences in the southern upstream part of the catchment (Muhar et al. 1996). The mountainous climate is alpine with low temperatures and a high annual rainfall.

Downstream from Lake Geneva, the average discharge of the Rhône River is 340 m^3^ s^−1^. Within the French part of the Rhône catchment (90,000 km^2^), the studied rivers belong to three main sub-catchment types. The alpine tributaries (R-ALPS), situated on the Rhône’s left eastern bank, flow from the highest alpine mountains. They comprised the upper Rhône tributaries (upstream from Lyon), the river Isère (11,890 km^2^, 333 m^3^ s^−1^) and the upper part of the Durance catchment. The hydrological regime is both influenced by rainfall and snowmelt due to the high elevation of these catchments and the dominance of a mountainous climate. The Rhône valley tributaries (R-VALL), the most important of which are the rivers Ain (3,765 km^2^, 123 m^3^ s^−1^), Ardèche (2,376 km^2^, 65 m^3^ s^−1^) and Drôme (1,663 km^2^, 20 m^3^ s^−1^), join the Rhône along its course. Their catchments are mountainous but with a hydrological regime linked to rainfall and a Mediterranean-influenced climate for the southern rivers. Near its mouth, the Rhône river discharge is 1,701 m^3^ s^−1^ with a pluvial regime, whereas the streamflow regime of the Upper-Rhône (upstream from the Saone River confluence) remains influenced by snowmelt. Coming from the north, the Saône River (R-SAON) is the largest tributary of the Rhône River (29,950 km^2^, 400 m^3^ s^−1^). The catchment comprises sedimentary plains surrounded by medium-sized mountains. The climate is continental and the streamflow regime depends on rainfall. Conversely to the three others areas, R-SAON is characterized by a lower elevation and the dominance of low to moderate river bed slopes.

### Historical fish fauna data

Among the different types of written fish historical sources, historical maps are one of the most informative because (1) they are based on fish surveys conducted by experts educated in biology and aimed at depicting the occurrence of all fish species, and (2) the species occurrences and species range limits are located on precise river sections of a few kilometers in length (Haidvogl et al. 2013a). These fish maps are of great interest as they enable analyzing with good accuracy the spatial distribution of species and their habitat requirements during industrialization, prior to the construction of the large dams and hydropower plants (Carrel 2002) and just after the end of the little ice age.

Two main sources were used to describe the occurrences of fish species at the turn of the twentieth century in D-SALZ (Fig. 1): the Kollmann’s map (1898) and the Fishery Cadastre of the Federal State Salzburg (1904). Information sources were questionnaires completed by the responsible fishing right owners and administrative authorities. They provided information about 26 occurring fish species for the river segments within their responsibility (Haidvogl et al. 2015).

Concerning the three areas within the Rhône catchment, the historical occurrences of fish species were obtained from 13 district or catchment fish maps (Fig. 1) drawn from 1910 to 1956 by Louis Léger (1866–1948) and his collaborators, providing information about 45 fish species (Carrel 2002): six maps for R-ALPS (1910,1910,1913, 1931, 1934,1942), four maps for R-VALL (1927, 1945, 1954, 1955), three maps for R-SAON (1924, 1927, 1945).

By the end of the nineteenth century, extensive civil engineering had already been carried out on Alpine tributaries to provide protection against erosion damage and flooding, and on the Rhone to improve navigation. No large hydroelectric dams, however, were operating on the Rhone itself, with few exceptions downstream of Lake Geneva (Bravard and Petts 1996).

**Fig. 2:**
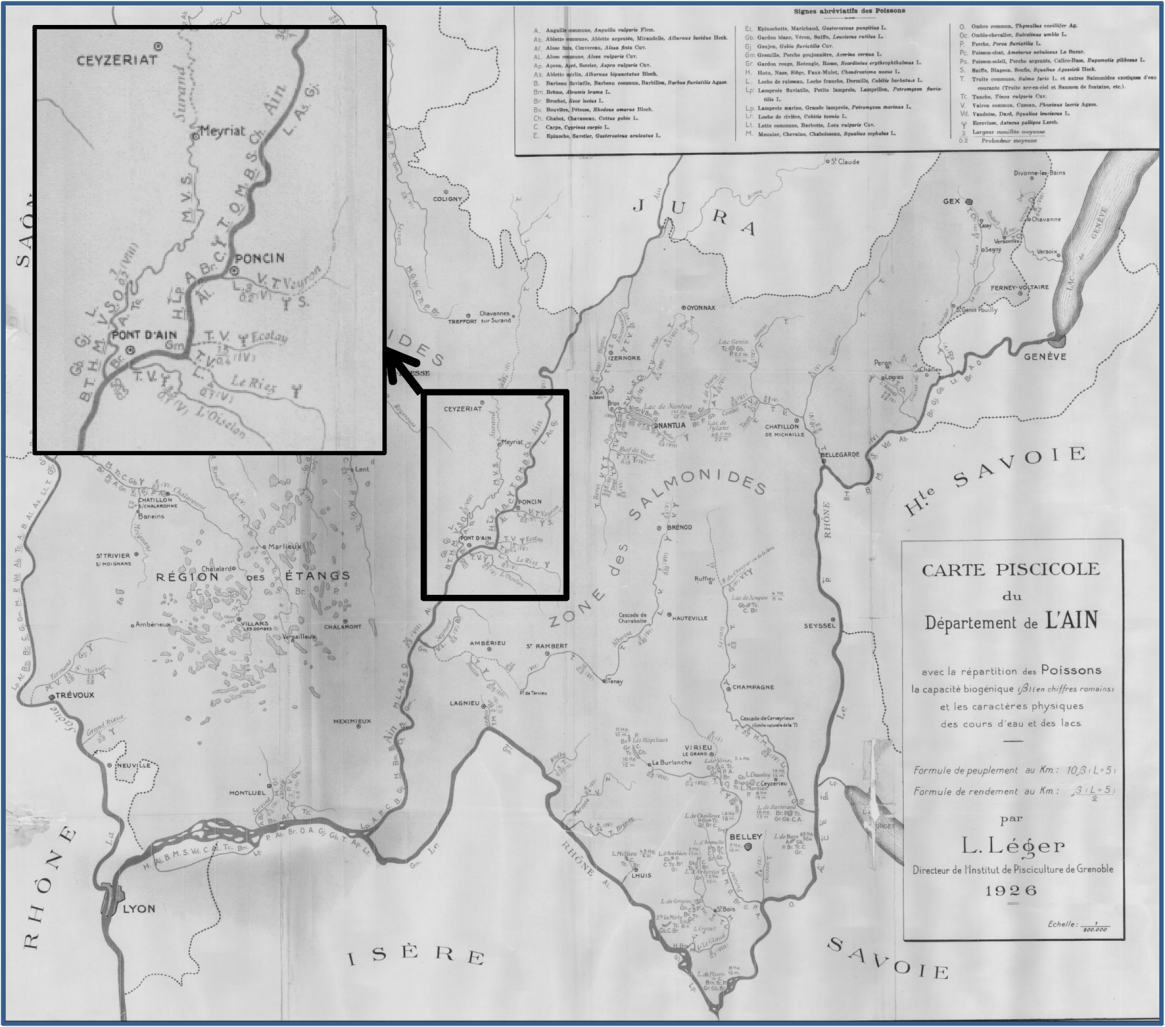
Example of an historical map of the Rhône catchment: the Ain district map, upstream from the town of Lyon (Léger 1927). Details from the area around the town of Pont d’Ain, along the course of the river Ain. The species names are indicated by *capital letters*

All these maps provided the list of species and their location in the different river segments of the hydrographic network (Fig. 2). For our analyses, fish species were identified based on current taxonomy (Kottelat and Freyhof 2007). Most fish species could be determined without doubt, but the identification of some at the species level is not absolutely certain (e.g. *Alburnus alburnus, Leuciscus leuciscus*, Petromyzontidae).

### Associated environmental variables

We considered only river segments with an upstream drainage area greater than 10 km^2^. The length of the river segments increased from upstream (7.1 km median length for upstream drainage area less than 50 km^2^) to downstream (23.7 median length for upstream drainage area over 1,000 km^2^). A GIS (Arc-GIS) and digital river networks were used to delineate all river segments defined on the historical maps and the associated historical fish occurrences (Austrian National River Network, French BD Carthage river network).

In order to predict historical fish species occurrences using the SDM from Logez et al. (2012), we estimated for each river segment UDA, STP, TJUL and TDIF. By intersecting the digital river network with digital elevation models (Rhône: 250 m resolution, Salzach: 10 m resolution), we defined UDA for each river segment, the elevation at the two extremities (of each river segment) and the river bed slope. For D-SALZ, UDA was obtained from the CCM-river network (v. 2.1; de Jager and Vogt 2010). For the Rhône catchment, the air temperature and precipitation values were extracted from the Tyndall data center (spatial resolution 10 × 10 min, Mitchell et al. 2004) for the periods corresponding to the establishment of the different fish maps. For D-SALZ, the air temperature and precipitation values were extracted from the Austrian historical temperature database of Chimany et al. (2013) (spatial resolution 5 min) for the period corresponding to Kollmann’s map (1891–1899).

A GIS layer was created for each monthly or yearly period for, respectively, air temperature and precipitation. For each river segment and for a considered time period, the mean monthly air temperatures were obtained by intersecting the corresponding temperature layers with the digital river network. The intersection between the temperature/precipitation layers and the layer defining the limit of the upstream catchment of each of the river segments allowed computing the mean annual values of temperature and precipitation for each UDA. The final parameters describing the climatic conditions (TJU and TDIF) were averaged over the 10 years before the edition of the historical maps.

### Testing SDM for predicting historical observations

We focused on the 14 fish species common to the Rhône and the Salzach catchments. We predicted their past occurrences using Logez et al. (2012) SDM and environmental conditions prevailing in the past; these were compared with historical observations. The models’ goodness of fit was estimated by area under the receiver operating characteristic curve, (AUC), sensitivity (% presence correctly predicted), specificity (% absence correctly predicted) and the overall correct classification rate (Fiedling and Bell 1997; Hosmer and Lemeshow 2000). AUC does not require transformation of the probability to binary data, and we used this criterion to evaluate the performance of the 14 models. AUC values range from 0 to 1, where 0.5 indicates that the model’s discrimination is no better than random sorting and 1 indicates that the model discriminates perfectly (Swets 1988). The application of these 14 SDM on our historical data was a “true” external validation and the AUC’s values were compared to the AUC values of the cross validation realized by Logez et al. (2012) on present data.

### Observed vs expected occurrences at the regional scale

There is a general scarcity of information at the local scale. Accordingly, examination of species occurrences across the watershed by combining historical observations yields a better understanding of the influence of human activities on the composition of the fish fauna at a given historical period (Harding et al. 1998). To evaluate the ability of the SDM to estimate the historical occurrences of each species for all sites and at the sub-catchment scale (D-SALZ, R-ALPS, R-VALL, R-SAON), we computed the cumulated observed presences (OBS) and the cumulated expected probabilities (EXP). For each species and each river segment, we computed the confidence interval (CI 95 %) around the expected probability value and, by summing up all the lower and the upper CI limits, we tested the significance (5 % level) of the difference between OBS and EXP; i.e. if OBS was within the range of the cumulated confidence interval.

To compare the quality of our predictions at the scale of each of the four areas, we regressed the cumulated observed occurrences (OBS) of the 14 species on their cumulated expected probabilities (EXP). If the whole assemblage of a given area was correctly predicted, we expected that the coefficient of determination of the regression would be significant, that the intercept of the regression line would not be significantly different from zero, and that the slope would not differ from one (Oberdorff et al. 2001).

### Long-term past and future species occurrences

We compared the past variability of species occurrences (1810–1999) in D-SALZ with the potential future variability (2000–2069) by computing the 260 annual predicted probabilities of presence for each river segment. From 1810 to 1999, we used Chimany et al. (2013) to reconstruct the annual evolution of the climate variables. To examine the effect of climate change on the distribution of species in the future, we used the Tyndall Centre for Climate Change Research Set of Scenarios (Mitchell et al. 2004). Potential future distributions (2000–2069) were predicted by the climate expected under SRES scenario A1 (very rapid economic growth, a global population that peaks in mid-century, and rapid introduction of new and more efficient technologies). Future climatic data were derived and averaged from three global circulation models (GCM), namely HadCM3 (Gordon et al. 2000), CGCM2 (Flato and Boer 2001), CSIRO-Mk2 (Hirst et al. 2000). We limited our predictions to the first two-thirds of the twenty first century so as not to apply the SDM outside of the range of the predictors used to calibrate the models. The associated uncertainties were estimated by computing the ratio between the mean difference between upper and lower limits of CI at 95 % and the mean predicted probability within a given time period. Results are presented for the species showing the most contrasted temporal pattern.

## Results

Within the 936 rivers segments considered (Table 1), 58 species were mentioned for a total number of 3,831 historical occurrences. The 14 studied species represented 68.6 % of these occurrences (D-SALZ: 91 %, Rhône catchment: 65 %) and they were mainly representative of three of the classical river types (Huet 1959): from upstream to downstream, the trout zone (*Salmo trutta, Cottus gobio, Phoxinus phoxinus, Barbatula barbatula*), the grayling zone (*Thymallus thymallus*) and the barbel zone (*Barbus barbus, Chondrostoma nasus, L. leuciscus, Lota lota,* *Squalius cephalus*). In contrast, species commonly inhabiting downstream river segments (bream zone) were underrepresented (*A. alburnus, Esox lucius, Perca fluviatilis, Rutilus rutilus*) due to the low number of river segments belonging to this last river type.

**Table 1:** Environmental characteristics within the four studied areas: median and minimal-maximal values between brackets

Areas	N	Elevation	UDA	SLOPE	TJUL	TDIF	Pann
R-ALPS	240	710 (134–1,379)	63.5 (10.0–12,860)	24.7 (0.2–174.7)	14.33 (5.8–18.7)	15.5 (11.3–17)	1,058 (764–1,427)
R-VALL	316	417 (64–1,103)	39.9 (10.0–69,720)	26.1 (0.4–200.6)	16.5 (12.7–19.7)	14.4 (13–17.5)	796 (690–1,124)
D-SALZ	206	867 (327–2,154)	35.9 (10.1–3,381)	37.3 (1.1–276.9)	14.1 (7.1–17.4)	18 (14.8–22)	1,479 (1,202–1,819)
R-SAON	174	259 (165–594)	65.7 (10.2–27,930)	4.5 (0.1–72.5)	16.5 (14.6–17.9)	14.2 (13.6–17.3)	805 (743–1,065)

*UDA* upstream drainage area,* SLOPE* river slope,* TJUL* monthly temperature in July,* TDIF* annual temperature range,* Pann* annual precipitation

### SDM performance on historical data

Predictive performances of the models (Table 2) were considered as good to excellent for nine species (AUC > 0.8), acceptable for four species (0.7 < AUC < 0.8, *S. trutta, P. phoxinus, B. barbatula, T. thymallus*) and poor for *C. gobio* (AUC close to 0.5). For this last species, the sensitivity was in particular very low. For the 13 remaining species, there was no clear tendency toward a higher specificity than sensitivity (respective mean values 0.80 and 0.78). AUC estimated based on historical data were greater than AUC estimated on the present data used to calibrate SDM excepted for *C. gobio* and *S. trutta* (Table 2).

**Table 2:** Goodness-of-fit statistics of the species distribution models applied to the historical data: species prevalence (Prev), relative occurrence in % (% Occ), area under the receiver operating characteristic curve (AUC), sensitivity (Sens), specificity (Spec) and overall correct classification (OCC)

Species	Prev	% Occ	AUC	Sens	Spec	OCC	AUC^a^
*Alburnus alburnus*	83	8.9	0.901	0.843	0.803	0.807	0.804
*Barbatula barbatula*	225	24.0	0.782	0.831	0.653	0.696	0.659
*Barbus barbus*	105	11.2	0.881	0.800	0.828	0.825	0.859
*Chondrostoma nasus*	76	8.1	0.858	0.789	0.851	0.846	0.830
*Cottus gobio*	304	32.5	0.464	0.132	0.889	0.643	0.703
*Esox lucius*	125	13.4	0.873	0.864	0.764	0.778	0.761
*Leuciscus leuciscus*	88	9.4	0.880	0.932	0.748	0.765	0.780
*Lota lota*	40	4.3	0.902	0.850	0.886	0.885	0.741
*Perca fluviatilis*	86	9.2	0.876	0.826	0.780	0.784	0.744
*Phoxinus phoxinus*	362	38.7	0.736	0.638	0.739	0.700	0.627
*Rutilus rutilus*	81	8.7	0.908	0.877	0.796	0.803	0.764
*Salmo trutta fario*	737	78.7	0.711	0.682	0.698	0.686	0.764
*Squalius cephalus*	242	25.9	0.869	0.851	0.741	0.769	0.726
*Thymallus thymallus*	73	7.8	0.745	0.630	0.803	0.790	0.718

^a^AUC obtained by Logez et al. (2012) with a cross-validation exercise using the same models with present data

### Historical fish species distribution at the catchment level

When considering all the studied sites (Table 3), the sum of the observed historical occurrences of five species were within the limits of the confidence intervals at 95 % of EXP, and could thus be considered as correctly estimated (*B. barbatula, L. lota, P. fluviatilis, R. rutilus and T. thymallus*). The presence of *A. alburnus*, *Esox Lucius* and of the four large rheophilic cyprinids *C. nasus, L. leuciscus,* *S. cephalus* and *B. barbus,* were underestimated by the models, especially the three last species. For these six last species, the results were similar when considering the four studied areas separately, except for R-VALL for *E. lucius*. Conversely, *S. trutta* was the only species overestimated by the models when considering all sites and three of the four areas separately (correct estimation for R-ALPS). For the other species, the ability of the SDM to correctly predict the historical species occurrences varied between the four studied areas (Table 3).

**Table 3:** Ability of the 14 species-specific models to predict the cumulated observed historical species prevalence for all sites and within each of the four studied areas (D-SALZ, R-ALPS, R-VALL, R-SAON): cumulated observed prevalence (OBS), cumulated expected probabilities (EXP) and 95 % confidence interval associated (CI)

Species	All sites		D-SALZ		R-ALPS		R-VALL		R-SAON	
	OBS	EXP [CI 95 %]	OBS	EXP [CI 95 %]	OBS	EXP [CI 95 %]	OBS	EXP [CI 95 %]	OBS	EXP [CI 95 %]
*Alburnus alburnus*	83	23.4 [13.7–38.4]	6	2.3 [1.3–4.3]	9	3.3 [1.8–6.3]	30	10.9 [6.6–16.5]	38	6.9 [4.0–11.3]
*Barbatula barbatula*	225	212.2 [168.7–268.8]	7	24.4 [17.1–35.8]	30	35.7 [25.9–51.0]	100	85.2 [69.9–103.6]	88	66.8 [55.9–78.5]
*Barbus barbus*	105	18.1 [9.7–36]	10	1.4 [0.6–3.8]	30	3.1 [1.7–7.0]	45	10.2 [6.0–17.6]	20	3.3 [1.5–7.5]
*Chondrostoma nasus*	76	10.4 [4.4–25.6]	14	0.5 [0.1–2.2]	9	1.9 [0.9–4.4]	28	7.1 [3.1–15.8]	25	0.8 [0.2–3.1]
*Cottus gobio*	304	332.4 [270.5–407.5]	81	58.9 [43.9–79.0]	117	69.7 [53.0–92.2]	34	121.7 [102.7–142.6]	72	82.1 [70.9–93.6]
*Esox lucius*	125	63.6 [45.0–90.7]	22	5.2 [3.3–8.5]	19	9.6 [5.9–16.1]	19	25.3 [18.3–34.7]	65	23.5 [17.5–31.3]
*Leuciscus leuciscus*	88	60.9 [42.7–87.0]	3	2.3 [1.3–4.4]	17	7.2 [4.5–12.2]	12	29.1 [21.2–39.5]	56	22.3 [15.8–31.0]
*Lota lota*	40	39.1 [22.4–67.3]	7	8.6 [5.4–13.9]	7	9.6 [5.0–17.9]	7	11.7 [6.6–20.2]	19	9.2 [5.4–15.3]
*Perca fluviatilis*	86	62.0 [45.1–86.0]	4	4.2 [2.7–6.9]	10	8.4 [5.5–13.1]	17	28.8 [21.8–38.1]	55	20.6 [15.1–27.9]
*Phoxinus phoxinus*	362	288.4 [236.1–353.1]	42	40.1 [29.4–55.4]	45	56.9 [42.8–76.6]	170	112.5 [95.3–131.6]	105	78.9 [68.6–89.5]
*Rutilus rutilus*	81	81.6 [60.7–110.0]	3	4.5 [2.9–7.3]	12	11.1 [7.5–16.8]	17	37.5 [28.9–48.4]	49	28.5 [21.4–37.4]
*Salmo trutta fario*	737	878.7 [852.1–896.1]	183	197.7 [192.0–200.8]	223	228.9 [220.7–233.3]	240	295.5 [288.7–300.8]	91	156.6 [150.7–161.2]
*Squalius cephalus*	242	82.3 [62.1–109.1]	18	6.8 [4.5–10.3]	47	12.1 [8.6–17.6]	93	39.5 [31.0–50.0]	84	23.9 [18.1–31.2]
*Thymallus thymallus*	73	106.6 [68.3–169.4]	47	28.6 [18.3–45.8]	12	25.0 [15.1–43.6]	9	33.5 [21.9–51.1]	5	19.5 [13.1–28.9]

In D_SALZ and R-ALPS, OBS and EXP did not differ for five and six species, respectively. But the occurrences of only three and one species were correctly predicted in R-VALL and R-SAON, respectively. In R-SAON, most of the species were underestimated, except for *S. trutta* and *T. thymallus* (overestimation).

**Fig. 3:**
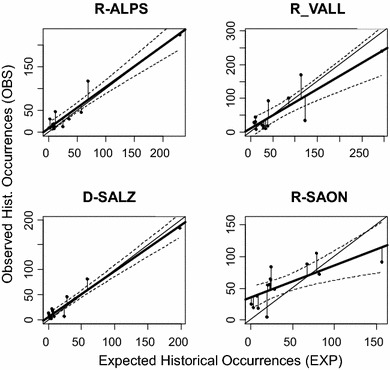
Plots of expected (sum of probability values) vs historically observed occurrence values of the 14 species (*black dots*) for the four areas (D-SALZ, R-ALPS, R-VALL, R-SAON). Confidence interval limits (95 %, *hatched lines*) around the regression lines (*bold solid lines*). Regression line of slope 1 and intercept 0 (*solid line*)

The regressions (Fig. 3) of the 14 species-specific OBS on the corresponding EXP were highly significant for D-SALZ, R-ALPS and R_VALL (*p* < 0.001), but only at *p* < 0.01 for R-SAON (Table 4). The determination coefficients were excellent for R-ALPS and D-SALZ (over 0.91) but lower for R-VALL (0.706). For R-SAON, only 50 % of the variability of OBS was explained by EXP. The intercept and the slope of the regression lines were not statistically different from respectively zero and one (Table 4) for D-SALZ, R-ALPS and R-VALL. But these two parameters differed significantly from, respectively, zero and one for R-SAON.

**Table 4:** Regressions of the sum of the observed occurrences on the sum of the predicted occurrences for each of the four studied areas: determination coefficient (R^2^), intercept (between brackets: significance of Student *t* test against zero), intercept (between brackets: significance of Student *t* test against one)

Areas	R^2^	Intercept	Slope
D-SALZ	0.951 (*p* < 0.001)	6.702 (ns)	0.916 (ns)
R-ALPS	0.914 (*p* < 0.001)	9.065 (ns)	0.953 (ns)
R-VALL	0.736 (*p* < 0.001)	11.728 (ns)	0.774 (ns)
R-SAON	0.506 (*p* < 0.01)	35.460 (*p* < 0.001)	0.508 (*p* < 0.01)

### Long-term variability

**Fig. 4:**
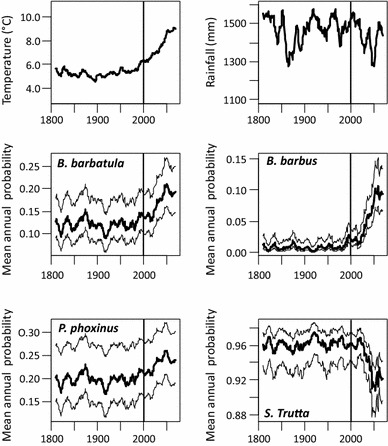
Evolution of the mean annual air temperature, the mean annual rainfall (simple moving averages over the previous 10 years), and the expected variability of four species occurrences within the Salzach catchment (D-SALZ) from 1810 to 2069: *B. barbatula*, *B. barbus*, *P. phoxinus* and *S. trutta*. Mean annual probability of presence (*bold solid line*) and associated confidence interval (95 %, *grey lines*). From 2000 on, the considered climate values are those from the IPCC scenario A1

The reconstruction of the potential temporal variability of the species’ mean annual probabilities of occurrence enables comparing the past and the potential future species distribution (Fig. 4). The mean annual rainfall (all mean values as moving averages over the previous 10 years) were similar during the nineteenth and twentieth centuries (1,447 and 1,490 mm, respectively), as were the mean air temperatures (4.48 and 4.69 °C, respectively). As a consequence, the STP values were quite similar during these two last centuries. From 2000 to 2069, the mean air temperature would increase by 2.69 °C, rising to 8.4 °C. From 2010 on, the fish have been and will continue experiencing, each year, a mean air temperature always higher than maximum air temperature recorded during the last two centuries (5.64 °C). In contrast, the range of the mean annual rainfall expected during the first half of the twenty first century is comparable to the range observed during the nineteenth and the twentieth century (1,273–1,576 and 1,280–1,555 mm, respectively).

For the past period (1810–1999), the mean annual expected probabilities and their range for all segments were as follows: *B. barbus* 0.008 (0.002–0.028), *B. barbatula* 0.121 (0.090–0.155), *P. phoxinus* 0.198 (0.166–0.230) and *S. trutta* 0.961 (0.949–0.975). The associated uncertainties were very high for *B. barbus*, high for *B. barbatula* and *P. phoxinus*, and limited for *S. trutta*.

Under the climatic scenario used in this study, the mean probabilities for the period 2000–2069 were 0.053 (*B. barbus*), 0.168 (*B. barbatula*), 0.232 (*P. phoxinus*) and 0.942 (*S. trutta*) with associated uncertainties of 1.01, 0.58, 0.50 and 0.04, respectively. *B. barbus* probability increased regularly and significantly until 2050 in relation with the temperature change. *P. phoxinus* also tended to increase its prevalence, but the associated CI showed that this species remained in its previous range of variability. The increase of *B. barbatula* was clearer, but the overlap between the CI before and after 2000 was large. *S. trutta* decreased quite clearly with a more limited CI overlap between past and future conditions. Finally, only *B. barbus* and *S. trutta* showed a clear potential change in their probability of occurrence in the Salzach catchment within the next century.

## Discussion

In this paper, we predicted historical fish species occurrences from the nineteenth century to the two-thirds mark of the twenty first century using present-data-based SDM and compared these expected values with historically observed occurrences from four different sub-catchments located in France and Austria.

### SDM performances for historical data

Our results highlight the good predictive performance of the SDM on historical data for 13 of the 14 studied species. For all of them except two, the AUC values associated with the comparison between prediction and historical observations are higher than AUC values obtained by Logez et al. (2012) with a cross-validation. In addition, the present data used to calibrate the models and the historical occurrences were not obtained using similar sampling techniques. Present species occurrences were obtained using classical electro-fishing techniques either by wading or by boat, depending on stream depth (Pont et al. 2006). Historical occurrences were based on fish surveys conducted by experts educated in sciences and in collaboration with professional fishermen.

A major classical problem with such historical data is that, most of the time, absence is not really registered (i.e. mainly presence-only data). In our study, this does not seem to be the case. For the 13 species correctly predicted by the models, the sensitivity was in general comparable to specificity, and we conclude that our historical maps yield accurate information about both species presence and absence. This contrasts with many other historical sources (fisheries sources, fish trading sources, fish consumption description), which typically focused only on particular species of commercial interest (Haidvogl et al. 2013a). The good performances of the present-data-based models tend to demonstrate that, in the case of such historical fish maps, the quality of the historical information provided by these experts is comparable to that obtained from classical electrofishing sampling. Nevertheless, as demonstrated by Lahoz-Montfort et al. (2014), the imperfect detection of species occurrence can have serious consequences for the efficiency of SDM, and the quality of presence-absence data remains a major objective for species distribution modelling.

A potential limit of our work was the type of statistical models we used: more recent techniques are considered to outperform the traditional GLM (Buisson et al. 2010). Among other methodological limits, SDM are very sensitive to collinearity between predictor variables (Graham 2003). Collinearity has huge effects on prediction quality as soon as the collinearity between the predictor variables is modified or if the model is used to predict species’ distributions in new geographic regions or under changed climatic conditions. Dormann et al. (2013), however, demonstrated that more recent methods specifically designed for collinearity did not outperform classical GLM with respect to sensitivity to collinearity variation. Moreover, Lahoz-Montfort et al. (2014) recently demonstrated that presence-background methods are equally affected by false absence as the classical presence-absence method. Logez et al. (2012) also showed that the model response to different ecological gradients was in agreement with the known ecology of the considered species, a point that is as important as model performance (Austin 2007). Finally, the ability to associate a confidence interval to the expected probability provides the opportunity to more precisely evaluate the quality of the fit between the model and the observed occurrences at different scales (see below).

### Shifting baseline reference conditions

Beyond the classical analysis of models, examining species-specific OBS and EXP at the regional level (sub-catchment) allows interpreting discrepancies between observed and predicted historical species distributions within the four areas. The seven species showing the same relationships between OBS and EXP for each of the four areas are of primary interest.

The four large cyprinids (*C. nasus, L. Leuciscus,* *S. cephalus* and *B. barbus*) were always underestimated by our current-data-based SDM. These species are large rheophilic cyprinids inhabiting medium-sized to large rivers (barbel zone). *B. Barbus*, *C. nasus* and *S. cephalus* are potamodromic (Noble et al. 2007) and undergo migration for reproduction within the tributaries or the floodplain (Kirchhofer 1996; Schiemer 2000). Since the late nineteenth century, most of rivers have been channelized, diked and disconnected from their previous floodplain (Petts 1984). In Austria, Haidvogl et al. (2013b), analyzing the historical distributions of *B. barbus* and *C. nasus* in the nineteenth century, showed that, nowadays, they occur in only 58 and 68 % of their past distributional area.

Similarly, *E. lucius* occurrences were also underestimated by SDM. This species lives in slow-flowing waters, migrates towards floodplains during high discharge periods for reproduction and has also been severely affected by channelization (Casselman and Lewis 1996). In addition, the afforestation of most of the alpine catchments since the end of the nineteenth century drastically reduced the coarse sediment load within the river network and induced a shift in the fluvial dynamics of rivers from aggradation to incision (Pont et al. 2009). This led to a continuous shrinkage of their associated floodplains and the favorable habitats for *E*. *lucius* reproduction (D-SALZ, R-ALPS). Our present-data-based SDMs were calibrated using a dataset of sites not or only slightly impacted by local anthropogenic pressures. This was done to describe a “reference situation”, following the minimally disturbed condition concept (Stoddard et al. 2006). Nevertheless, the quasi-absence of medium-sized to large rivers unaffected by channelization and dike erection lead to the selection of a calibration dataset of river segments where the consequences of these long-term hydromorphological modifications of rivers on the fish communities cannot be easily noticed. In the case of these four species (*C. nasus, B. barbus, S. cephalus, E. lucius*), the shifting baseline concept is applicable to describe this shift towards the description of current reference condition differing from those prevailing in rivers before the late nineteenth century.

### Past historical anthropogenic alteration of fish assemblages

In contrast, the brown trout *S. trutta* was overestimated by SDM within each of the four studied areas. It is also the only species for which AUC estimated based on historical data were lower than AUC estimated on the present data used to calibrate SDM. For almost a century, the European brown trout populations have been largely affected by stocking because of fishery interest (Bagliniere 1991). This is one potential explanation for its increased occurrence nowadays compared to the nineteenth century. In addition, this species is classified as highly intolerant to human disturbances (stenothermic, oxygen depletion intolerant and reproducing only in fast-flowing habitat); it is underrepresented when degraded ecological conditions prevail (Pont et al. 2006; Noble et al. 2007). Such an interpretation could also be considered for *T. thymallus*, another well-known intolerant species in the Rhône catchment (R-ALPS, R-VALL, R-SAON). The overestimation of the occurrence of these two species could then be related to historical anthropogenic alterations of the studied rivers.

Such a possible impact of anthropogenic pressures can be analyzed by comparing the ability of our SDM to correctly predict the historical fish assemblages between the four studied areas. The high value of the determination coefficients of the regressions between OBS and EXP for D-SALZ and R-ALPS demonstrated that our models were able to correctly describe the structure of the fish assemblages in these two areas. Forecasted fish assemblages were probably not far from the fish assemblage that could be expected in the absence of significant local anthropogenic pressures. In contrast, the lower quality of the linear regressions for R-VALL and R-SAON could be interpreted as an impact of anthropogenic disturbances affecting the fish assemblage (Oberdorff et al. 2001). In addition, in the case of R-SAON, the significant differences of the slope and the intercept of the regression line from zero and one highlight the important deviation of the fish assemblage from the expected one in the absence of major human disturbance. At the end of the nineteenth century, the R-VALL and R-SAON catchments were characterized by high human densities and the dominance of cropland, whereas the two alpine areas D-SALZ and R-ALPS were less human-impacted (Goldewijk et al. 2010). The historical maps describing the R-SAON area reveal the presence of numerous ponds and canals, and several cases of water pollution are described (Hesse and Paris 1924). Such altered ecological conditions could explain the SDM underestimation of the occurrences of the two tolerant and slow-flowing species *P. fluviatilis* and *R. rutilus* in R-VALL and R-SAON (Noble et al. 2007), whereas the observed occurrences of the intolerant species *S. trutta* and *T. thymallus* are much lower than expected. Finally, note that four of the seven fish maps from R-VALL and R-SAON have been edited after the Second World War. In contrast, the D_SALZ map and three of the six fish maps for R-ALPS were edited before the end of the First World War. Accordingly, our results demonstrate the increase of anthropogenic impacts and the degradation of fish communities during the first half of the twentieth century.

### Influence of climate variability on long-term past and future species distribution

The comparison of the past expected temporal variability of the species prevalence and the associated uncertainties with the potential evolution under the applied climate scenario in the next century allows a more comprehensive discussion of the SDM prediction for the future. The prevalence of only two of the four species tested seemed to significantly change in D-SALZ (*B. barbus* and *S. trutta*). This agrees with previous results in the literature: a decrease of cold to cool-water species and the expansion of cyprinids (Buisson et al. 2008; Comte and Grenouillet 2013). Nevertheless, in the two other cases (*B. barbatula, P. phoxinus*), the uncertainties associated with the past occurrences were too large in relation to the expected future trends. Moreover, the temporal variability during the two last centuries is not trivial. For example, the prevalence of *B. barbatula* was 57 % higher during the warmer and low rainfall period 1871–1877 than during the colder and high rainfall period 1915–1922 (mean July temperature of 12.96 versus 14.8 °C, mean annual rainfall of 1,554 versus 1,338 mm). These results highlight the need to consider the past variability of species distribution when analyzing future trends in relation with global warming scenarios and to consider the uncertainties associated with SDM predictions (Buisson et al. 2010).

## Conclusion

The comparison between observed and predicted historical fish species distribution in four different European areas demonstrated the utility of SDM in reconstructing past fish assemblages and their long-term changes. Uncertainties associated with SDM predictions were very useful to assess the efficiency of these models. In general, the efficiency of SDM based on current fish sampling in minimally disturbed river segments to describe historical reference conditions is acceptable, especially for the upstream part of the river networks (trout zone, grayling zone). Nevertheless, for those species depending on the main channel but also on floodplain water bodies, the comparison between predicted and historically observed data showed a clear shifting baseline in reference conditions. In fact, very few naturally functioning floodplain rivers remain in Europe (Górski et al. 2011), limiting the possibilities to describe current undisturbed fish communities from large rivers. In practice, the use of the reference condition approach to assess rivers in Europe is adequate. Nonetheless, one has to consider that minimally disturbed conditions can only be taken into consideration for the main channel of rivers.

One could argue that reference conditions based on historical observations are more efficient. Historical data, however, do not always reflect undisturbed conditions. Our results highlight that fish assemblages were seriously altered in several areas by human activities at the turn of the twentieth century. The nineteenth century was already a period of intense human pressures within most European catchments, reflecting the dense human population, deforestation, agriculture, mining and the presence of industries (Pounds 1985; Bravard and Petts 1996; Pont et al. 2009). In addition, major changes in river morphology occurred in Europe long before the industrialization period. This, in particular, refers to deforestation and implementation of small structures (water mills) along the river course (Walter and Merritts 2008).

The long-term evolution of fish species distribution also depends on environmental variables acting at the large scale, such as climate. Past variability in temperature and rainfall during the two last centuries may have considerably modified the composition of fish assemblages, in particular during warmer and low rainfall periods. Using models to reconstruct the past variability of species distributions and their associated uncertainties is a major first step to more comprehensively understand the consequences of future global warming, and in particular to test whether species will be subjected to an environmental situation they never experienced in the past.

A more detailed analysis of the intensity of human pressures acting historically, and of the impact of climate variability, is necessary to confirm and detail our interpretations. Nonetheless, our results show the potential of SDM based on present “undisturbed sites” to analyze the historical ecological status of European rivers. In agreement with Tingley and Beissinger (2009), we claim that historical data are of primary importance to document fish range changes in relation to human activities, environmental modifications and global warming: the keepers of historical data have crucial roles in curating data and collaborating with aquatic ecologists.
